# Supplementary material for: Identification of photoperiod-induced specific miRNAs in the adrenal glands of Sunite sheep (Ovis aries)
Source: Front Vet Sci. 2022 Jul 22;9:888207. doi: 10.3389/fvets.2022.888207 (PMC9354845; doi:10.3389/fvets.2022.888207)
Supplement: Supplementary file 1 [file Data_Sheet_1.ZIP › Supplementary file/miRDeep2 file.zip!/miRDeep2 file/Know_Structure/LP42A1/oar-mir-200c.pdf]

miRBase precursor	: oar-mir-200c
Total read count	: 200
oar-miR-200c read count	: 200
remaining reads	: 0

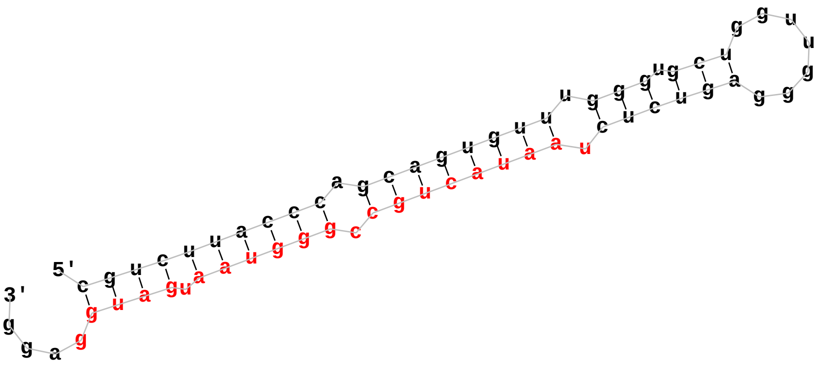

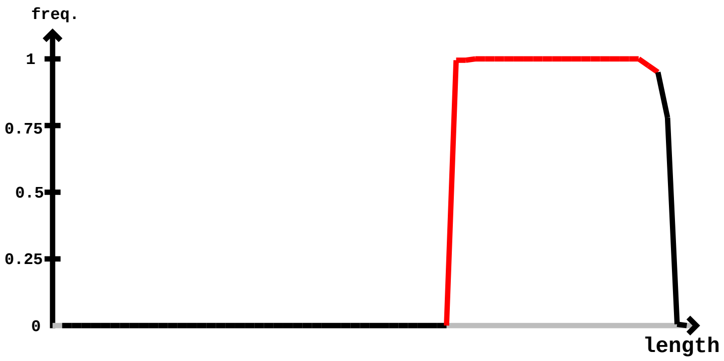


```
                                        oar-miR-200c
5'- cgucuuacccagcaguguuugggugcugguugggagucucuaauacugccggguaaugauggagg   -3'    exp
    ((((((((((.((((((((.(((.(((.......)))))).)))))))).)))))).))))....    reads      mm         sample
    ........................................uaauacugccggguaaugau.....    5          0          seq
    ........................................uaauacugccggguaaugaug....    5          0          seq
    ........................................uaauacugccggguaaugaugg...    34         0          seq
    ........................................uaauacugccggguaaugaugga..    154        0          seq
    ........................................uaauacugccggguaaugauggag.    1          0          seq
    ..........................................auacugccggguaaugaugga..    1          0          seq
```
